# Methyl 4-benz­yloxy-2-hy­droxy­benzoate

**DOI:** 10.1107/S1600536812046491

**Published:** 2012-11-17

**Authors:** B. S. Palakshamurthy, H. T. Srinivasa, Vijith Kumar, S. Sreenivasa, H. C. Devarajegowda

**Affiliations:** a Department of Physics, Yuvaraja’s College (Constituent College), University of Mysore, Mysore 570 005, Karnataka, India; b Raman Research Institute, C. V. Raman Avenue, Sadashivanagar, Bangalore 560 080, Karnataka, India; c Soild State and Structural Chemistry Unit, Indian Institute of Science, Bangalore 560 012, Karnataka, India; d Department of Studies and Research in Chemistry, Tumkur University, Tumkur 572 103, Karnataka, India

## Abstract

In the title compound, C_15_H_14_O_4_, the dihedral angle between the benzene rings is 67.18 (8)°. The C_a_—C_m_—O—C_a_ (a = aromatic and m = methyl­ene) torsion angle is 172.6 (3)° and an intra­molecular O—H⋯O hydrogen bond generates an *S*(6) ring. In the crystal, mol­ecules are linked by C—H⋯O hydrogen bonds into zigzag chains propagating in [001] and C—H⋯π inter­actions also occur.

## Related literature

For general background to benzyl­oxybenzoates, see: Pifferi *et al.* (1977 ▶); Ghosh *et al.* (2008 ▶). For related structures and further synthetic details, see: Tangdenpaisal *et al.* (2009 ▶); Kashi *et al.* (2010 ▶).
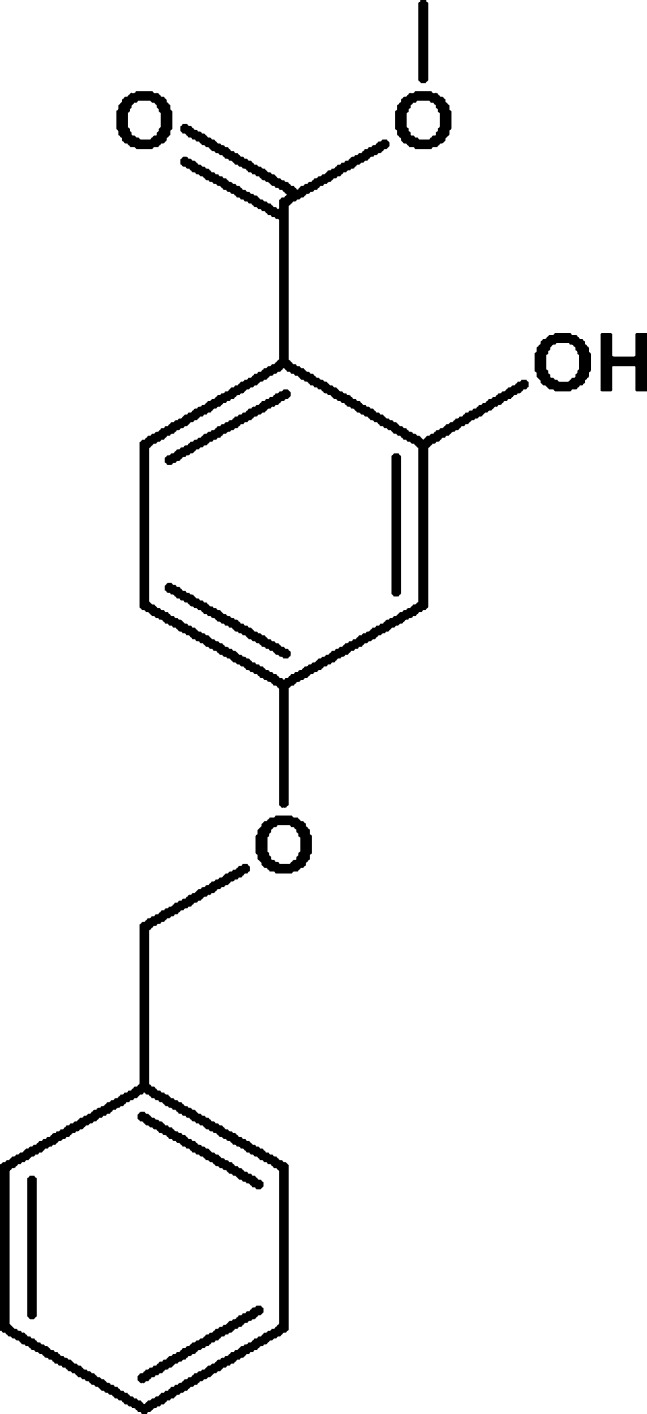


## Experimental

### 

#### Crystal data

C_15_H_14_O_4_
*M* *_r_* = 258.26
Triclinic, 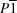

*a* = 5.7731 (10) Å
*b* = 7.9855 (14) Å
*c* = 14.046 (3) Å
α = 89.490 (6)°
β = 80.111 (5)°
γ = 87.210 (6)°
*V* = 637.16 (19) Å^3^
*Z* = 2
Mo *K*α radiation
μ = 0.10 mm^−1^
*T* = 293 K
0.24 × 0.22 × 0.18 mm

#### Data collection

Bruker SMART CCD diffractometer
Absorption correction: multi-scan (*SADABS*; Sheldrick, 2007 ▶) *T*_min_ = 0.977, *T*_max_ = 0.983
12770 measured reflections
2231 independent reflections
1679 reflections with *I* > 2σ(*I*)
*R*_int_ = 0.053

#### Refinement

*R*[*F* ^2^ > 2σ(*F* ^2^)] = 0.042
*wR*(*F* ^2^) = 0.129
*S* = 1.07
2231 reflections
173 parameters
H-atom parameters constrained
Δρ_max_ = 0.17 e Å^−3^
Δρ_min_ = −0.15 e Å^−3^

### 

Data collection: *SMART* (Bruker, 2001 ▶); cell refinement: *SAINT* (Bruker, 2001 ▶); data reduction: *SAINT*; program(s) used to solve structure: *SHELXS97* (Sheldrick, 2008 ▶); program(s) used to refine structure: *SHELXL97* (Sheldrick, 2008 ▶); molecular graphics: *ORTEP-3* (Farrugia, 2012) ▶; software used to prepare material for publication: *SHELXL97*.

## Supplementary Material

Crystal structure: contains datablock(s) I, global. DOI: 10.1107/S1600536812046491/hb6982sup1.cif

Structure factors: contains datablock(s) I. DOI: 10.1107/S1600536812046491/hb6982Isup2.hkl

Supplementary material file. DOI: 10.1107/S1600536812046491/hb6982Isup3.cml

Additional supplementary materials:  crystallographic information; 3D view; checkCIF report

## Figures and Tables

**Table 1:** Hydrogen-bond geometry (Å, °) *Cg*1 is the centroid of the C5–C10 ring.

*D*—H⋯*A*	*D*—H	H⋯*A*	*D*⋯*A*	*D*—H⋯*A*
O2—H2⋯O3	0.82	1.89	2.6133 (17)	146
C19—H19*C*⋯O2^i^	0.96	2.54	3.469 (3)	163
C11—H11*A*⋯*Cg*1^ii^	0.97	2.78	3.5991 (19)	143
C13—H13⋯*Cg*1^iii^	0.93	2.95	3.7324 (19)	142

Symmetry codes: (i) 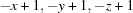; (ii) 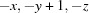; (iii) 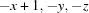.

**Fig. 1.**
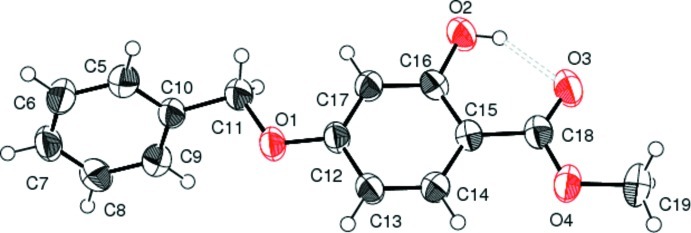
The molecular structure of the title compound. Displacement ellipsoids are drawn at the 50% probability level.

**Fig. 2.**
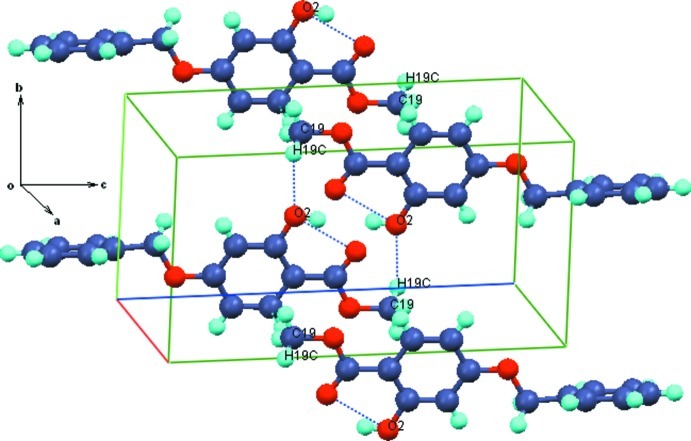
The packing of molecules.

## supplementary crystallographic information

### Comment

Protected benzyloxyphenols and benzyloxybenzoates are an important class of compounds for the syntheses of pharmaceuticals, natural products and polymers (Pifferi *et al.*, 1977). In particular, Methyl 4-(benzyloxy)-2-hydroxybenzoates are essential components of various types of liquid crystals and materials science (Ghosh *et al.*, 2008), also will play an important role in synthesizing rod shaped liquid crystal which exhibits monotropic nematic mesophase (Kashi *et al.*, 2010). Solid supported reagents have been employed for the protection and deprotection process frequently in organic synthesis particularly for title compound (Tangdenpaisal *et al.*, 2009).

The asymmetric unit of the title compound is shown in Fig. 1. The dihedral angle between the least-squares planes of the two benzene rings (C5–C10) and (C12–C17) is 67.18 (8)°. The crystal structure is characterized by intermolecular C19—H19···O2 and intramolecular O2—H2···O3 hydrogen bonding and also features C11—H11A···C_g_(1) (C5–C10) and C13—H13···C_g_(1) (C5–C10) (Table 1) interactions.

### Experimental

A mixture of methyl 2,4-dihydroxybenzoate (1 mmol) and benzylchloride (1.2 mmol) anhydrous potassium carbonate (1.5 mmol) in methyethylketone was refluxed for 12hrs. After completion of reaction, the reaction mixture was extracted into chloroform washed with water dried over anhydrous sodium sulfate. On removal of organic solvent offered crude white solid, which was purified by column chromatography on silica gel (60–120 mesh size) using 5% ethyl acetate in hexane as an eluent. Finaly the title compound was recrystallized from pure ethanol to yield colourless plates.

### Refinement

All H atoms were positioned geometrically, with C—H = 0.93 Å for aromatic H, C—H = 0.97 Å for methylene H and C—H = 0.96 Å for methyl H,and refined using a riding model with *U*_iso_(H) = 1.5*U*_eq_(C) for methyl H and *U*_iso_(H) = 1.2*U*_eq_(C) for all other H.

### Crystal data

**Table:**

C_15_H_14_O_4_	*Z* = 2
*M**_r_* = 258.26	*F*(000) = 272
Triclinic, *P*1	*D*_x_ = 1.346 Mg m^−^^3^
Hall symbol: -P 1	Melting point: 377 K
*a* = 5.7731 (10) Å	Mo *K*α radiation, λ = 0.71073 Å
*b* = 7.9855 (14) Å	Cell parameters from 2231 reflections
*c* = 14.046 (3) Å	θ = 2.6–25.0°
α = 89.490 (6)°	µ = 0.10 mm^−^^1^
β = 80.111 (5)°	*T* = 293 K
γ = 87.210 (6)°	Plate, colourless
*V* = 637.16 (19) Å^3^	0.24 × 0.22 × 0.18 mm

### Data collection

**Table:**

Bruker SMART CCD diffractometer	2231 independent reflections
Radiation source: fine-focus sealed tube	1679 reflections with *I* > 2σ(*I*)
Graphite monochromator	*R*_int_ = 0.053
ω and φ scans	θ_max_ = 25.0°, θ_min_ = 2.6°
Absorption correction: multi-scan (*SADABS*; Sheldrick, 2007)	*h* = −6→6
*T*_min_ = 0.977, *T*_max_ = 0.983	*k* = −9→9
12770 measured reflections	*l* = −16→16

### Refinement

**Table:**

Refinement on *F*^2^	Secondary atom site location: difference Fourier map
Least-squares matrix: full	Hydrogen site location: inferred from neighbouring sites
*R*[*F*^2^ > 2σ(*F*^2^)] = 0.042	H-atom parameters constrained
*wR*(*F*^2^) = 0.129	*w* = 1/[σ^2^(*F*_o_^2^) + (0.0713*P*)^2^ + 0.0386*P*] where *P* = (*F*_o_^2^ + 2*F*_c_^2^)/3
*S* = 1.07	(Δ/σ)_max_ < 0.001
2231 reflections	Δρ_max_ = 0.17 e Å^−^^3^
173 parameters	Δρ_min_ = −0.15 e Å^−^^3^
0 restraints	Extinction correction: *SHELXL97* (Sheldrick, 2008), Fc^*^=kFc[1+0.001xFc^2^λ^3^/sin(2θ)]^-1/4^
Primary atom site location: structure-invariant direct methods	Extinction coefficient: 0.069 (9)

### Special details

**Table:**

Geometry. All s.u.'s (except the s.u. in the dihedral angle between two l.s. planes) are estimated using the full covariance matrix. The cell s.u.'s are taken into account individually in the estimation of s.u.'s in distances, angles and torsion angles; correlations between s.u.'s in cell parameters are only used when they are defined by crystal symmetry. An approximate (isotropic) treatment of cell s.u.'s is used for estimating s.u.'s involving l.s. planes.
Refinement. Refinement of *F*^2^ against ALL reflections. The weighted *R*-factor *wR* and goodness of fit *S* are based on *F*^2^, conventional *R*-factors *R* are based on *F*, with *F* set to zero for negative *F*^2^. The threshold expression of *F*^2^ > 2σ(*F*^2^) is used only for calculating *R*-factors(gt) *etc*. and is not relevant to the choice of reflections for refinement. *R*-factors based on *F*^2^ are statistically about twice as large as those based on *F*, and *R*- factors based on ALL data will be even larger.

### Fractional atomic coordinates and isotropic or equivalent isotropic displacement parameters (Å^2^)

**Table:**

	*x*	*y*	*z*	*U*_iso_*/*U*_eq_	
O1	0.3228 (2)	0.20059 (14)	0.10019 (7)	0.0518 (4)	
O2	0.1364 (2)	0.41392 (14)	0.42117 (8)	0.0574 (4)	
H2	0.1856	0.4109	0.4726	0.086*	
O3	0.4189 (2)	0.31994 (16)	0.54049 (8)	0.0605 (4)	
O4	0.7269 (2)	0.14432 (15)	0.48726 (8)	0.0568 (4)	
C5	−0.0453 (3)	0.2119 (2)	−0.06754 (12)	0.0516 (5)	
H5	−0.1633	0.1609	−0.0253	0.062*	
C6	−0.0486 (3)	0.2107 (2)	−0.16596 (12)	0.0579 (5)	
H6	−0.1693	0.1600	−0.1893	0.069*	
C7	0.1252 (3)	0.2840 (2)	−0.22893 (12)	0.0563 (5)	
H7	0.1224	0.2838	−0.2949	0.068*	
C8	0.3032 (3)	0.3575 (2)	−0.19423 (12)	0.0578 (5)	
H8	0.4226	0.4063	−0.2368	0.069*	
C9	0.3056 (3)	0.3594 (2)	−0.09595 (12)	0.0549 (5)	
H9	0.4271	0.4097	−0.0730	0.066*	
C10	0.1305 (3)	0.2876 (2)	−0.03155 (11)	0.0443 (4)	
C11	0.1266 (3)	0.2994 (2)	0.07531 (11)	0.0534 (5)	
H11A	0.1366	0.4154	0.0935	0.064*	
H11B	−0.0197	0.2585	0.1101	0.064*	
C12	0.3616 (3)	0.21291 (19)	0.19290 (10)	0.0406 (4)	
C13	0.5597 (3)	0.1217 (2)	0.21350 (11)	0.0477 (4)	
H13	0.6521	0.0552	0.1662	0.057*	
C14	0.6165 (3)	0.1310 (2)	0.30389 (11)	0.0471 (4)	
H14	0.7493	0.0707	0.3171	0.057*	
C15	0.4801 (3)	0.22901 (18)	0.37723 (10)	0.0400 (4)	
C16	0.2802 (3)	0.31707 (18)	0.35554 (11)	0.0406 (4)	
C17	0.2211 (3)	0.30868 (19)	0.26383 (11)	0.0433 (4)	
H17	0.0875	0.3674	0.2503	0.052*	
C18	0.5349 (3)	0.23696 (19)	0.47507 (11)	0.0444 (4)	
C19	0.7991 (4)	0.1564 (2)	0.58079 (13)	0.0637 (5)	
H19A	0.9378	0.0855	0.5817	0.096*	
H19B	0.6749	0.1213	0.6303	0.096*	
H19C	0.8323	0.2704	0.5922	0.096*	

### Atomic displacement parameters (Å^2^)

**Table:**

	*U*^11^	*U*^22^	*U*^33^	*U*^12^	*U*^13^	*U*^23^
O1	0.0604 (7)	0.0624 (7)	0.0333 (6)	0.0172 (6)	−0.0155 (5)	−0.0058 (5)
O2	0.0632 (8)	0.0671 (8)	0.0402 (7)	0.0173 (6)	−0.0085 (6)	−0.0148 (5)
O3	0.0718 (9)	0.0731 (8)	0.0362 (7)	0.0062 (7)	−0.0110 (6)	−0.0084 (6)
O4	0.0628 (8)	0.0695 (8)	0.0423 (7)	0.0048 (6)	−0.0230 (6)	−0.0020 (6)
C5	0.0496 (10)	0.0602 (10)	0.0461 (10)	−0.0027 (8)	−0.0109 (8)	0.0012 (8)
C6	0.0567 (11)	0.0682 (12)	0.0534 (11)	0.0005 (9)	−0.0229 (9)	−0.0096 (9)
C7	0.0670 (12)	0.0686 (11)	0.0345 (9)	0.0143 (9)	−0.0164 (8)	−0.0038 (8)
C8	0.0580 (11)	0.0699 (12)	0.0427 (10)	−0.0022 (9)	−0.0012 (8)	0.0039 (8)
C9	0.0491 (10)	0.0693 (12)	0.0484 (10)	−0.0070 (9)	−0.0130 (8)	−0.0057 (8)
C10	0.0454 (9)	0.0522 (9)	0.0358 (8)	0.0082 (7)	−0.0114 (7)	−0.0019 (7)
C11	0.0523 (10)	0.0702 (11)	0.0378 (9)	0.0136 (8)	−0.0127 (8)	−0.0024 (8)
C12	0.0471 (9)	0.0443 (9)	0.0312 (8)	0.0008 (7)	−0.0095 (7)	−0.0004 (6)
C13	0.0510 (10)	0.0563 (10)	0.0340 (8)	0.0119 (8)	−0.0060 (7)	−0.0059 (7)
C14	0.0457 (9)	0.0555 (10)	0.0401 (9)	0.0090 (8)	−0.0102 (7)	0.0008 (7)
C15	0.0446 (9)	0.0425 (8)	0.0333 (8)	−0.0026 (7)	−0.0077 (7)	0.0009 (6)
C16	0.0464 (9)	0.0393 (8)	0.0346 (8)	0.0000 (7)	−0.0035 (7)	−0.0026 (6)
C17	0.0452 (9)	0.0459 (9)	0.0396 (9)	0.0057 (7)	−0.0115 (7)	−0.0001 (7)
C18	0.0507 (10)	0.0461 (9)	0.0377 (9)	−0.0071 (8)	−0.0094 (7)	0.0026 (7)
C19	0.0778 (13)	0.0748 (12)	0.0459 (10)	−0.0062 (10)	−0.0311 (9)	0.0036 (9)

### Geometric parameters (Å, º)

**Table:**

O1—C12	1.3638 (17)	C9—H9	0.9300
O1—C11	1.4393 (19)	C10—C11	1.501 (2)
O2—C16	1.3509 (18)	C11—H11A	0.9700
O2—H2	0.8200	C11—H11B	0.9700
O3—C18	1.2198 (19)	C12—C17	1.382 (2)
O4—C18	1.337 (2)	C12—C13	1.397 (2)
O4—C19	1.4502 (19)	C13—C14	1.368 (2)
C5—C10	1.376 (2)	C13—H13	0.9300
C5—C6	1.386 (2)	C14—C15	1.403 (2)
C5—H5	0.9300	C14—H14	0.9300
C6—C7	1.369 (3)	C15—C16	1.399 (2)
C6—H6	0.9300	C15—C18	1.465 (2)
C7—C8	1.369 (3)	C16—C17	1.391 (2)
C7—H7	0.9300	C17—H17	0.9300
C8—C9	1.383 (2)	C19—H19A	0.9600
C8—H8	0.9300	C19—H19B	0.9600
C9—C10	1.379 (2)	C19—H19C	0.9600
			
C12—O1—C11	116.82 (12)	O1—C12—C17	124.30 (14)
C16—O2—H2	109.5	O1—C12—C13	115.41 (14)
C18—O4—C19	116.31 (14)	C17—C12—C13	120.29 (14)
C10—C5—C6	120.76 (17)	C14—C13—C12	119.41 (15)
C10—C5—H5	119.6	C14—C13—H13	120.3
C6—C5—H5	119.6	C12—C13—H13	120.3
C7—C6—C5	120.18 (16)	C13—C14—C15	121.85 (15)
C7—C6—H6	119.9	C13—C14—H14	119.1
C5—C6—H6	119.9	C15—C14—H14	119.1
C8—C7—C6	119.67 (16)	C16—C15—C14	117.79 (14)
C8—C7—H7	120.2	C16—C15—C18	119.60 (14)
C6—C7—H7	120.2	C14—C15—C18	122.57 (14)
C7—C8—C9	120.09 (17)	O2—C16—C17	116.75 (14)
C7—C8—H8	120.0	O2—C16—C15	122.45 (14)
C9—C8—H8	120.0	C17—C16—C15	120.81 (14)
C10—C9—C8	120.92 (16)	C12—C17—C16	119.83 (14)
C10—C9—H9	119.5	C12—C17—H17	120.1
C8—C9—H9	119.5	C16—C17—H17	120.1
C5—C10—C9	118.37 (15)	O3—C18—O4	122.01 (14)
C5—C10—C11	121.05 (16)	O3—C18—C15	124.12 (15)
C9—C10—C11	120.52 (15)	O4—C18—C15	113.87 (14)
O1—C11—C10	109.23 (13)	O4—C19—H19A	109.5
O1—C11—H11A	109.8	O4—C19—H19B	109.5
C10—C11—H11A	109.8	H19A—C19—H19B	109.5
O1—C11—H11B	109.8	O4—C19—H19C	109.5
C10—C11—H11B	109.8	H19A—C19—H19C	109.5
H11A—C11—H11B	108.3	H19B—C19—H19C	109.5

### Hydrogen-bond geometry (Å, º)

Cg1 is the centroid of the C5–C10 ring.

**Table:**

*D*—H···*A*	*D*—H	H···*A*	*D*···*A*	*D*—H···*A*
O2—H2···O3	0.82	1.89	2.6133 (17)	146
C19—H19*C*···O2^i^	0.96	2.54	3.469 (3)	163
C11—H11*A*···*Cg*1^ii^	0.97	2.78	3.5991 (19)	143
C13—H13···*Cg*1^iii^	0.93	2.95	3.7324 (19)	142

Symmetry codes: (i) −*x*+1, −*y*+1, −*z*+1; (ii) −*x*, −*y*+1, −*z*; (iii) −*x*+1, −*y*, −*z*.
